# Shannon Entropy of a Hydrogenic Impurity on a Conical Surface: Confinement and Aharonov–Bohm Effects

**DOI:** 10.3390/e28030356

**Published:** 2026-03-22

**Authors:** Luis Manuel Arvizu, Eleuterio Castaño, Norberto Aquino

**Affiliations:** Departamento de Física, Universidad Autónoma Metropolitana-Iztapalapa, Av. Ferrocarril de San Rafael Atlixco 186, Col. Leyes de Reforma 1ª Sección, Iztapalapa, Mexico City 09310, Mexico; arvizu13@xanum.uam.mx (L.M.A.); ele@xanum.uam.mx (E.C.)

**Keywords:** energy eigenvalues, eigenfunctions, quantum cone, Shannon entropy, Aharonov–Bohm magnetic field

## Abstract

In this work, we solve the Schrödinger equation for a hydrogenic impurity located at the apex of a right circular cone, with the electron constrained to move on the conical surface of semi-aperture angle $\theta_{0}$ and subjected to an Aharonov–Bohm magnetic flux along the symmetry axis. Analytical expressions for the energy eigenvalues and normalized radial wave functions are obtained in terms of the principal quantum number *n* and the angular quantum number m, the magnetic flux ν, and the cone angle. The Shannon entropy is evaluated in both configuration and momentum spaces for several low-lying states, and its variation with ν and $\theta_{0}$ is analyzed in detail. When the magnetic flux vanishes, pairs of states $\left(n,\;m\right)$ and $\left(n,\;-m\right)$ share the same entropic behavior; for finite flux, this degeneracy is lifted and the entropies depend explicitly on the state, the cone geometry, and the flux strength. Finally, we verify that the entropic sum $S_{r}+S_{p}$ fulfills the Bialynicki-Birula–Mycielski bound, providing an information-theoretic consistency check for the model.

## 1. Introduction

Spatial confinement and geometry play a central role in determining the physical properties of quantum confined systems. Low-dimensional structures allow for modification of the energy spectrum and wave functions via geometric constraints and external fields, making them an ideal framework for exploring quantum confinement effects and their possible applications. In this context, information theory provides powerful tools to analyze the spatial distribution and localization properties of quantum states, with applications ranging from the derivation of fundamental equations in physics [1] to communication theory [2,3], robot navigation [4], financial markets [5,6,7], data analysis [8,9], machine learning [10,11,12,13], seismology [14], image processing [15,16,17], biomedical imaging [18,19], cryptography [20], the study of early stone tools [21], and black holes [22].

In quantum physics, information-theoretic measures have become valuable tools for characterizing the localization, correlation, and complexity of electronic states in both free and confined systems. Among them, Shannon entropy is the most widely employed quantity [23,24,25,26,27,28,29,30,31,32,33,34,35,36,37,38,39,40,41,42,43,44,45,46,47,48,49,50], having been applied to high-dimensional harmonic and hydrogenic systems [23], models with Dirac delta [24] and Pöschl–Teller potentials [25], mean excitation energy [26], and the analysis of chemical bonding and electron delocalization [27,28,29,30,31]. It has also been used to assess basis-set quality [28,29,30], to quantify correlation in wave functions [32,33], and in the study of avoided crossing atomic levels due to laser interaction [34] and the Shannon entropy of spatially confined hydrogen atoms subjected to a Hellmann potential [35]. More recently, Shannon entropy has been employed to interpret spectroscopic trends along the hydrogen isoelectronic sequence [36] and to compare molecular structures through information-based descriptors [37]. A substantial body of work has also explored spatially confined quantum systems from an information-theoretic viewpoint [38,39,40,41,42,43,44,45,46,47,48,49,50,51]. These studies include hydrogenic and helium atoms [38,39,41,42,46,47,48], many-electron systems [40], and simple models such as a particle in a circular box [43,44,45]. Attention has been paid to situations in which external magnetic fields or Aharonov–Bohm flux modify the energy spectrum and the spatial localization of wave functions [52,53,54,55]. In this context, Olendski [52,53] analyzed a quantum ring in uniform and Aharonov–Bohm magnetic fields using Shannon entropy, Fisher information, disequilibrium, and complexity measures, while other works have examined thermodynamic and informational effects of Aharonov–Bohm flux in different quantum systems [54,56].

Recently, the case of an electron confined to a finite region on the surface of a circular cone was studied as a function of the cone angular amplitude and the length of the confining region, with and without an Aharonov–Bohm magnetic field [50,51].

The study of quantum dots is of great importance from both a theoretical point of view and in terms of their technological applications. Some of their applications are infrared laser amplifiers, photodetectors, and optoelectronic modulators. In biology, they are used in fluorescence energy transfer and fluorescent labeling, to name but a few. Quantum dots of various geometries have been studied, including cylindrical [56], spherical [57], rings [58], ellipsoids [59], and cones [50,60,61]. The size and form of the quantum dots determine the wavelengths of its emission spectrum.

The energies, wave functions, and transitions between states of the hydrogenic atom on the surface of a cone are a newly studied system [62]. As the cone apex half-angle $\theta_{0}$ tends to π/2, the two-dimensional hydrogen atom is recovered, while the $\theta_{0}\to 0$ one-dimensional hydrogen atom is obtained. In general, when $\theta_{0}\neq \pi /2,$ a greater number of permitted electric-dipole transitions is obtained than in the two-dimensional case.

In this work, we study a hydrogenic atom on the surface of a right circular cone, which is also affected by an Aharonov–Bohm flux. We calculate the eigenenergies, eigenfunctions, and Shannon entropy in configuration and momentum spaces as functions of the angular aperture and the magnetic flux for different radial and angular quantum numbers.

This work is organized as follows: in Section 2, we obtain and solve the Schrödinger equation for a particle confined on the surface cone in curvilinear coordinates under an Aharonov–Bohm Field. In Section 3, we provide the theoretical framework for Shannon entropy in configuration and momentum space. The results and discussion of this work are provided in Section 4. Finally, in Section 5, we give our conclusions.

## 2. Theoretical Model: Eigenfunctions and Eigenenergies

We study a model hydrogen atom on the surface of a right-circular cylinder whose axis contains an infinitesimally thin solenoid with a magnetic flux Φ. The nucleus is fixed at the apex of the cone. At the same time, the electron moves on its surface under the effects of the Coulomb interaction with the nucleus and the magnetic vector potential of the solenoid, which introduces an Aharonov–Bohm effect since the magnetic field is zero outside the solenoid. The electron mass is $m_{o},$ and its electric charge −e; the electric charge of the nucleus is e.

**Figure 1:**
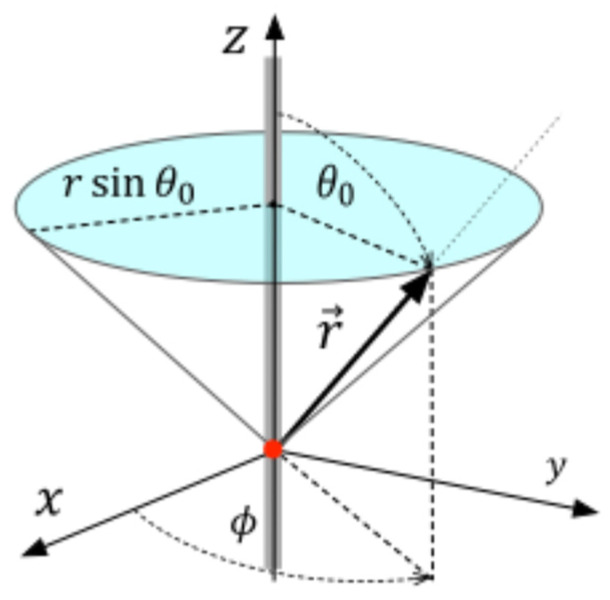
Right circular cone of angular semi-aperture $\theta_{0}$, 0≤r≤∞, 0≤ϕ<2π.

As shown in Figure 1, the origin of coordinates is placed at the cone apex, the *z*-axis lies along its symmetry axis, and the cone angular semi-aperture is $\theta_{0}$.

Since the motion of the electron is restricted to the conical surface, its position is entirely described by two coordinates: the radial distance from the origin r and the circumferential angle ϕ, and, therefore, the position vector is given by

(1) $$\vec{r}=r\hat{r},$$

where $\hat{r}$ is the radial unit vector in spherical coordinates,

(2) $$\hat{r}=\sin\theta_{0}\cos\phi \hat{x}+\sin\theta_{0}\sin\phi \hat{y}+\cos\theta_{0}\hat{z}\;$$

Then, we have that the differential vector position is

(3) $$d\vec{r}=dr\hat{r}+r\sin\theta_{0}d\phi \hat{\phi },$$

and, therefore, the differential area element is $dA=r\sin\theta_{0}drd\phi$.

The electrostatic potential energy due to the interaction between the nucleus and the electron is

(4) $$V\left(r\right)=-\frac{e^{2}}{4\pi \epsilon_{0}r\;}\;,$$

and vector potential, in the symmetric gauge, caused by the solenoid is

(5) $$\vec{A}\left(r,\phi \right)=\frac{\Phi }{2\pi \;r\;sin{\;\theta }_{0}}\hat{\phi }=A\left(r\right)\hat{\phi },$$

with $\hat{\phi }$ being the circumferential unit vector

(6) $$\hat{\phi }=-\sin\phi \hat{x}+\cos\phi \hat{y}.$$

With these potentials, we solved the time-independent Schrödinger equation,

(7) $$\hat{H}\psi \left(r,\phi \right)=E\psi \left(r,\phi \right),$$

where the Hamiltonian, in atomic units $m_{0}=\hbar =e=4\pi \epsilon_{0}=1$, is

(8) $$\hat{H}=-\frac{1}{2}\left[\frac{1}{r}\frac{\partial }{\partial r}\left(r\frac{\partial }{\partial r}\right)+\frac{1}{r^{2}{sin}^{2}\theta_{0}}{\left(\frac{\partial }{\partial \phi }-i\nu \right)}^{2}\right]-\frac{1}{r}.$$

with

(9) $$\nu =\frac{\Phi }{\Phi_{0}},$$

which is a dimensionless magnetic flux in units of quantum $\Phi_{0}=h/e$.

The solution is

(10) $$\psi \left(r,\phi \right)=R\left(r\right)\frac{e^{im\phi }}{\sqrt{2\pi }},\;\;m=0,\pm 1,\pm 2,\ldots$$

Substituting the separable form given in Equation (10) into the Schrödinger equation, one obtains the radial equation

(11) $$\frac{1}{r}\frac{d}{dr}\left(r\frac{dR\left(r\right)}{dr}\right)-\left[\frac{{\left(m+\nu \right)}^{2}}{{\left(r\;sin\theta_{0}\right)}^{2}}-\frac{2}{r}+2E\right]R\left(r\right)=0.$$

For bound states $\left(E<0\right)$, we set E=−ε/2, ε>0, and introduce a change in variable $x=\sqrt{\varepsilon }r$, and the parameter

(12) $$\mu =\frac{\left|m+\nu \right|}{\sin\theta_{0}}.$$

Using the ansatz $R(x)=x^{\mu }e^{-x}F(x)$, the radial equation is transformed into a confluent hypergeometric equation. Square-integrability of the radial wave function requires the hypergeometric series to terminate, which occurs only for a discrete set of solutions labeled by a non-negative integer $n_{r}=0,1,2,\ldots$

This quantization condition leads to

(13) $$E_{n_{r},m}=-\frac{1}{2{\left(n_{r}+\frac{\left|m+\nu \right|}{sin\theta_{0}}+\frac{1}{2}\right)}^{2}}.$$

Defining a principal quantum number n, the radial and principal quantum numbers are related by

(14) $$n=n_{r}+\left|m\right|+1,$$

with $\left|m\right|=0,1,2,\ldots ,n-1$; (13) is now written as

(15) $$E_{n,m}=-\frac{1}{2{\left(\left(n-\frac{1}{2}\right)+\frac{\left|m+\nu \right|}{sin\;\theta_{0}}-\left|m\right|\right)}^{2}}.$$

With this identification, the energy spectrum may be equivalently written in terms of n, while the radial wave function is naturally expressed through associated Laguerre polynomials of order $n-\left|m\right|-1=n_{r}$.

Accordingly, the normalized radial wave function can be written as

(16) Rn,mr=22μ+2εn,m n−m−1!sinθ0n−m−1+2μ!2n−2m−1+2μ12×e−εn,mrεn,mrμLn−m−12μ2εn,mr,

where ε=−2E>0 for bound states.

Please note that for $\theta_{0}=\pi /2$ and ν=0, the problem is reduced to the two-dimensional hydrogen atom, which was studied by Yang [63] et al. and Aquino and Castaño [64], among other authors.

The radial probability density is given by

(17) $$\rho_{n,m}\left(r\right)dr=\int_{\phi =0}^{2\pi }{\left|\psi_{n,m}(r,\phi )\right|}^{2}sin\;\theta_{0}r\;dr\;d\phi ={\left|R_{n,m}\left(r\right)\right|}^{2}sin{\;\theta }_{0}r\;dr.$$

## 3. Shannon Entropy

In this section, we compute the Shannon entropy for different states as a function of the radial distance r, the cone semi–aperture $\theta_{0}$, and the magnetic flux ν. The squared radial wavefunction is introduced as

(18) $$\Phi_{n,m}\left(r\right)={\left|R_{n,m}(r,\phi )\right|}^{2}.$$

The two-dimensional Fourier transform on the conical surface is defined as

(19) $${\tilde{\psi }}_{n,m}\left(p,\vartheta \right)=\frac{1}{2\pi }\int_{r=0}^{\infty }\int_{\phi =0}^{2\pi }\psi_{n,m}(r,\phi )e^{-i\vec{p}\cdot \vec{r}}sin{\;\theta }_{0}rdrd\phi ,$$

where

(20) $$\vec{p}\cdot \vec{r}\equiv pr\cos\left(\vartheta -\phi \right),$$

and therefore,

(21) $${\tilde{\psi }}_{n,m}\left(p,\vartheta \right)=\frac{i^{m}e^{im\vartheta }}{\sqrt{2\pi }}\int_{0}^{\infty }R_{n,m}\left(r\right)J_{\mu }\left(pr\right)\;sin{\;\theta }_{0}r\;dr,$$

This expression naturally identifies the radial part of the momentum–space wavefunction as

(22) $${\tilde{R}}_{n,m}\left(p\right)=\int_{0}^{\infty }R_{n,m}\left(r\right)J_{\mu }\left(pr\right)\;sin{\;\theta }_{0}r\;dr,$$

so that its squared modulus defines

(23) $${\tilde{\Phi }}_{n,m}(p)={\left|{\tilde{R}}_{n,m}\left(p\right)\right|}^{2}.$$

The Shannon entropy $S_{r}$ in configuration space is given by

(24) $$S_{r}\left(n,m\right)=-\int_{r=0}^{\infty }\int_{\phi =0}^{2\pi }\Phi_{n,m}\left(r\right)ln\left(\Phi_{n,m}\left(r\right)\right)sin{\;\theta }_{0}r\;dr\;d\phi ,$$

which is a functional of the probability density that measures the dispersion of the probability density throughout the confining region in a global manner.

In momentum space the Shannon entropy $S_{p}$ is given by

(25) $$S_{p}\left(n,m\right)=-\int_{p=0}^{\infty }\int_{\vartheta =0}^{2\pi }{\tilde{\Phi }}_{n,m}(p)ln\left({\tilde{\Phi }}_{n,m}\left(p\right)\right)\frac{p}{sin\;\theta_{0}}dp\;d\vartheta .\;$$

The interpretation of $S_{p}$ is analogous to that of $S_{r}$ but now in momentum space.

It must be emphasized that these entropies must satisfy the bound established by Bialynicki-Birula and Mycielski [65], the BBM uncertainty principle:

(26) $$S_{t}=S_{r}+S_{p}\geq D\left(1+\ln\pi \right),$$

where *D* is the dimensionality of the system.

## 4. Results and Discussion

### 4.1. Energy Eigenvalues

**Figure 2:**
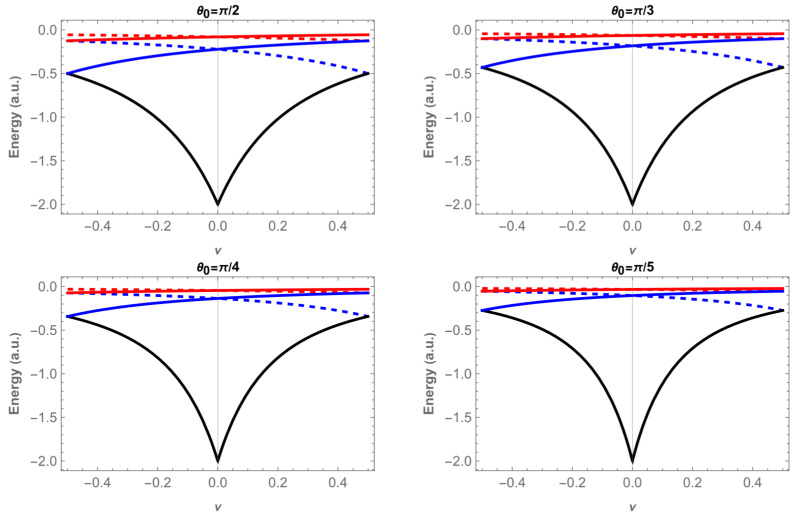
Energies, $E_{1,m}$ as a function of the magnetic flux ν for four different apex angles $\theta_{0}$. Continuous lines correspond to m=0 in black, m=1 in blue and, m=2 in red; dashed lines are for m=−2, in red, and for m=−1 in blue.

**Figure 3:**
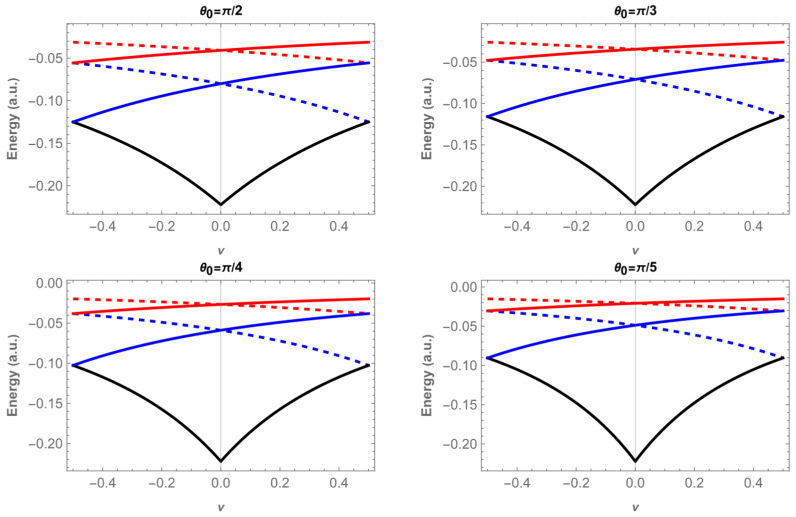
Energies, $E_{2,m}$ as a function of the magnetic flux ν for four different apex angles $\theta_{0}$. Continuous lines correspond to m=0 in black, m=1 in blue and, m=2 in red; dashed lines are for m=−2, in red, and for m=−1 in blue.

The energy eigenvalues are shown in Figure 2 and Figure 3 as a function of the magnetic flux ν, for the states $\left(1,m\right)$ and $\left(2,m\right)$, with m=0, ±1, ±2 and for four different values of $\theta_{0}$: π/2, when the system is confined to a plane, π/3, π/4 and π/5. The eigenenergies become less negative as $\theta_{0}$ decreases, in agreement with Heisenberg’s uncertainty principle: as the size of the confining region decreases, the electron linear momentum and kinetic energy must increase.

In Figure 2 the ground state $\left(n=1,m=0\right)$ is represented by the black curve. The energy curve is an even function of ν with a minimum at ν=0, and its value is independent of $\theta_{0}$, because for m=0 and ν=0 the denominator of the energy expression (Equation (15)) does not involve $sin{\;\theta }_{0}$. The other states with m=±1 (blue) and m=±2 (red) lie at higher energies. Their dependence on the flux is governed by $\left|m+\nu \right|$, so that the curves with +m shift upward for positive ν, while the corresponding −m partners shift upward for negative ν. In both cases, the eigenenergies become less negative as one moves away from ν=0.

For the states with n=2, the energies lie closer to zero (Figure 3), reflecting weaker binding compared to the states with n=1. The overall structure is similar, but the dependence on the flux is smoother, and the separation between different m values is reduced. This shows that the Coulomb interaction partly compensates the confinement effect, making these states less sensitive to both ν and $\theta_{0}$.

In both cases, degeneracies of order two appear at ν=0 and also at ν=0.5, since the condition $\left|m+\nu \right|$ = $\left|m^{\prime }+\nu \right|$ is satisfied (for instance, $\left(n,m\right)$ = $\left(1,0\right)$ and $\left(1,-1\right)$). These degeneracies are a general feature of Aharonov–Bohm [50] systems with cylindrical symmetry and persist for the hydrogenic impurity on a conical surface.

**Figure 4:**
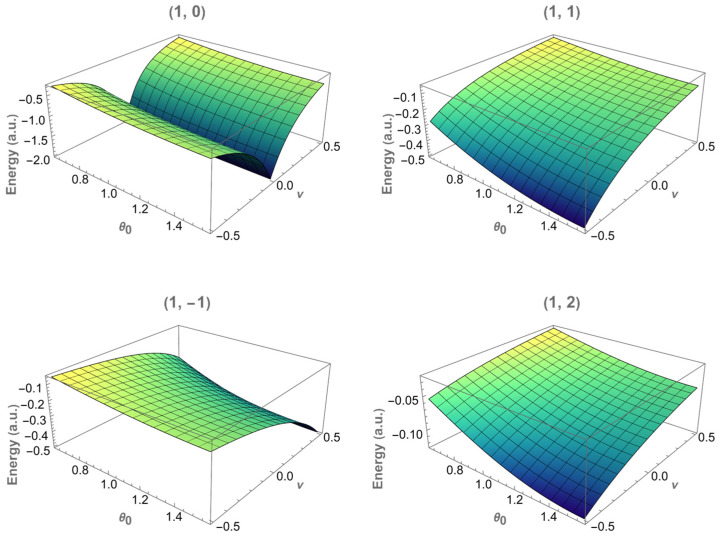
Energy surfaces $E_{1,m}(\nu ,\;\theta_{0})$ for the states with n=1 and magnetic quantum numbers m=0, ±1 and 2. Each panel corresponds to a fixed (n,m). The energy is shown as a function of the magnetic flux ν and the cone apex angle $\theta_{0}$.

**Figure 5:**
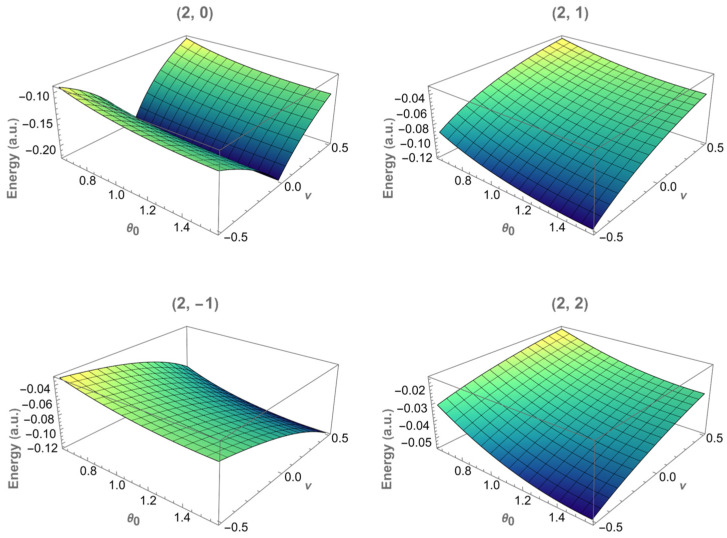
Energy surfaces $E_{2,m}(\nu ,\;\theta_{0})$ for the states with n=2 and magnetic quantum numbers m=0, ±1 and 2. Each panel corresponds to a fixed (n,m). The energy is shown as a function of the magnetic flux ν and the cone apex angle $\theta_{0}$.

The three-dimensional energy surfaces shown in Figure 4 and Figure 5 provide a complementary representation of the results displayed in Figure 2 and Figure 3. Instead of fixing the apex angle $\theta_{0}$, the energy $E_{n,m}$ is now visualized as a function of both the magnetic flux ν and the cone apex angle $\theta_{0}$ for each fixed (n,m) state.

These surfaces summarize, in a single plot, the trends already discussed in the two-dimensional analysis. In particular, the gradual increase in the energy as $\theta_{0}$ decreases, and the asymmetric dependence on the flux for the states with m≠0 is clearly recovered. No additional qualitative features arise in the three-dimensional representation, confirming that the spectrum’s behavior is smoothly governed by the combined effects of geometry and magnetic flux.

### 4.2. Radial Probability Density

**Figure 6:**
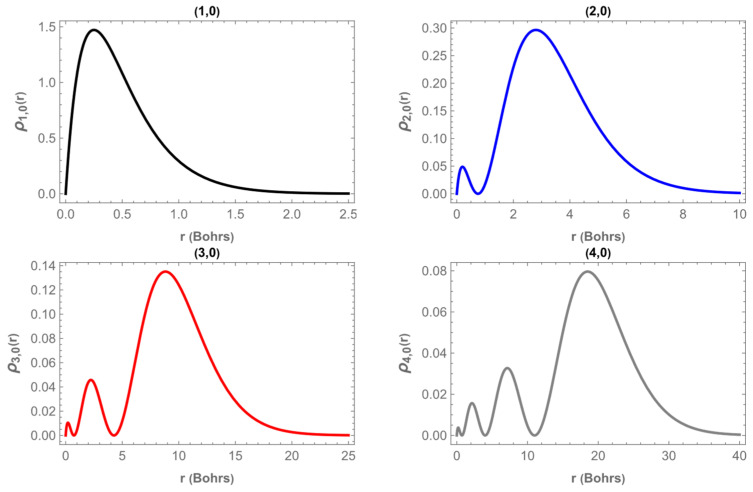
Radial distribution of the probability density, $\rho_{n,0}\left(r\right)$, as a function of *r* for ground state (1,0) and for (2,0), (3,0) and (4,0) states for an angle $\theta_{0}=\pi /2$ and ν=0.

Figure 6 shows the radial probability density $\rho_{n,0}\left(r\right)$, for the ground state $\left(n,m\right)$ = $\left(1,0\right)$ and for the three excited states, $\left(n,m\right)$ = $\left(2,0\right),\left(3,0\right)\;and\;\left(4,0\right)$ in the planar limit corresponding to $\theta_{0}=\pi /2$ and without magnetic flux, ν=0. The behavior of $\rho_{n,0}\left(r\right)$ exhibits the typical structure of hydrogen-like systems: the ground state displays a single maximum close to the origin and decays monotonically, whereas higher excited states develop additional radial nodes. As *n* increases, the first maximum of the distribution moves to larger r, and the overall profile becomes broader, indicating that the electron tends to occupy regions farther from the cone apex. This progressive outward shift in the maxima and the appearance of oscillations reflect the increasing spatial extension of the electron cloud as the excitation level grows. Therefore, Figure 6 provides a clear visualization of how the confinement weakens with increasing radial quantum number in the planar geometry.

**Figure 7:**
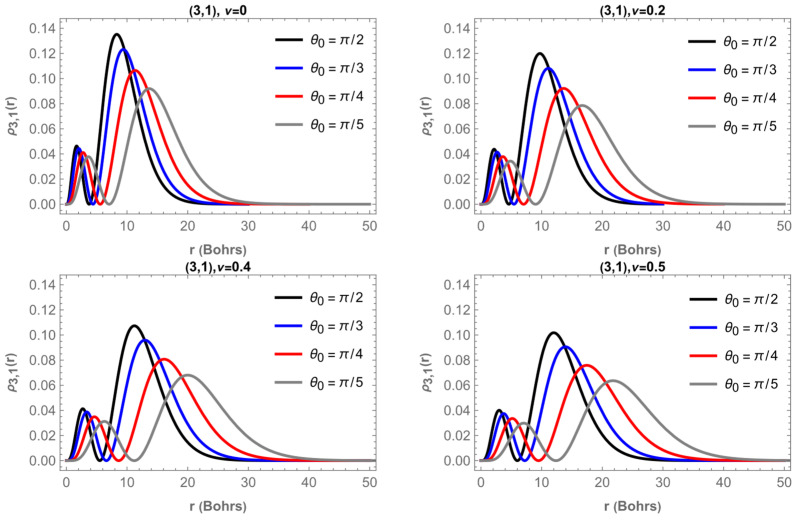
Radial probability density, $\rho_{3,1}\left(r\right)$, as a function of r for four different values of $\theta_{0}$ and ν.

Figure 7 shows the radial probability density $\rho_{3,1}\left(r\right)$ for the fixed state $\left(n,m\right)$ = $\left(3,1\right)$ as a function of the radius r. Each panel corresponds to a different value of the magnetic flux, specifically ν=0, 0.2, 0.4 and 0.5, while in each panel the four curves represent different apex angles $\theta_{0}=\pi /2,\;\pi /3,\;\pi /4\;and\;\pi /5$.

For the case without magnetic flux ν=0, the four curves reveal that the probability density broadens and its maximum moves toward larger r as the cone becomes narrower (i.e., as $\theta_{0}$ decreases). The most localized distribution corresponds to the planar limit, $\theta_{0}=\pi /2,$ whereas the smallest apex angle produces a more extended profile with the principal maximum farther from the apex.

As the magnetic flux increases to ν=0.2, the same geometric trend persists, but all the curves shift slightly outward and their peaks decrease in height, indicating that the presence of flux reduces the effective confinement. For ν=0.4, the displacement of the main peak becomes more pronounced; the inner lobe near the origin weakens and the outer lobe dominates, revealing that the probability density is redistributed toward larger r. Finally, at ν=0.5, the outward shift reaches its maximum: all angular cases show broader profiles and lower amplitudes, confirming that a stronger Aharonov–Bohm flux delocalizes the electron and decreases the probability of finding it near the cone apex.

In summary, the four panels demonstrate two consistent effects: (a) decreasing $\theta_{0}$ (narrower cone) produces a moderate outward shift and broadening of $\rho_{3,1}\left(r\right)$; and (b) increasing magnetic flux ν enhances that trend, pushing the entire distribution toward larger r, thus reducing its amplitude near the origin. The combination of both parameters controls the radial localization of the electron on the conical surface.

**Figure 8:**
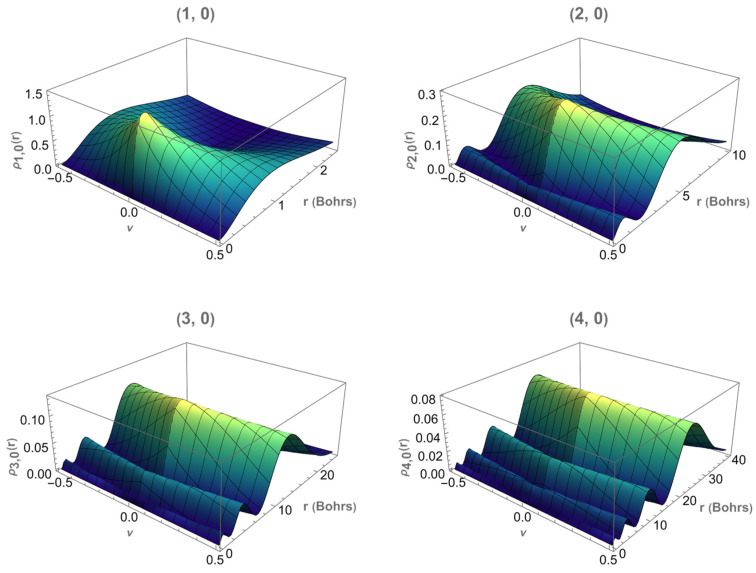
Three-dimensional representation of the radial probability density $\rho_{n,0}\left(r\right)$ as a function of the magnetic flux ν and the radial coordinate r, for the states (n,m)=(1,0),(2,0),(3,0) and (4,0) in the planar limit $\theta_{0}=\pi /2$.

The three-dimensional plots shown in Figure 8 provide a complementary visualization of the radial probability density $\rho_{n,0}\left(r\right)$, extending the results of Figure 6 by allowing the magnetic flux ν to vary. For each fixed (n,0) state, the density is represented as a function of the radial coordinate r and the flux ν.

The main features discussed in the two-dimensional case are preserved. In particular, the position of the radial maxima shifts outward as the principal quantum number n increases, and additional radial nodes appear for excited states. The dependence on the magnetic flux introduces a smooth deformation of the density profile but does not alter its overall structure.

This representation shows that the radial localization of the electron is primarily governed by the radial quantum number, while the magnetic flux modulates the distribution continuously without inducing qualitative changes in the nodal pattern.

Although the three-dimensional plots in Figure 8 are shown for the planar limit $\theta_{0}=\pi /2$, the behavior of the radial probability density for smaller apex angles can be inferred from the two-dimensional results. As $\theta_{0}$ decreases, the geometric confinement of the cone leads to a displacement of the radial probability density toward larger values of r. The main maxima move outward and the distribution becomes broader, indicating that the electron tends to occupy regions farther from the cone apex.

### 4.3. Shannon Entropy Results

#### 4.3.1. Shannon Entropy in Configuration Space

**Figure 9:**
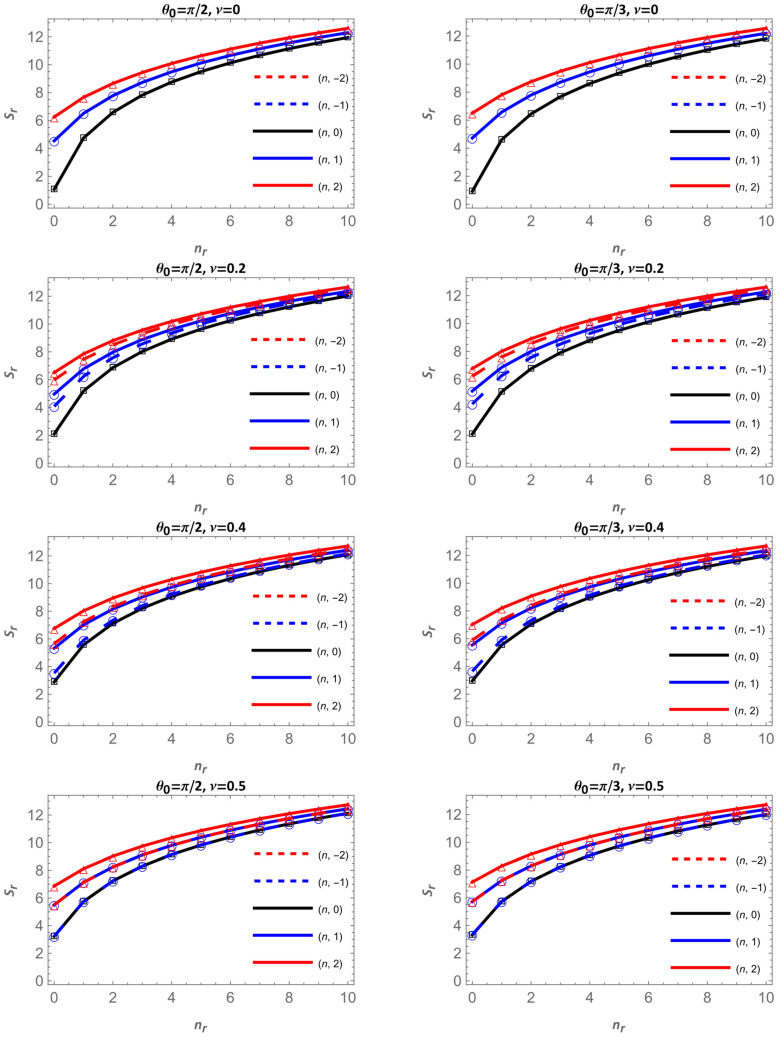
Shannon entropy in configuration space as a function of $n_{r}$ for $\theta_{0}=\pi /2\;and\;\pi /3$. Continuous lines correspond to m=0 in black, m=1 in blue and m=2 in red; dashed lines are for m=−2, in red, and for m=−1 in blue.

**Figure 10:**
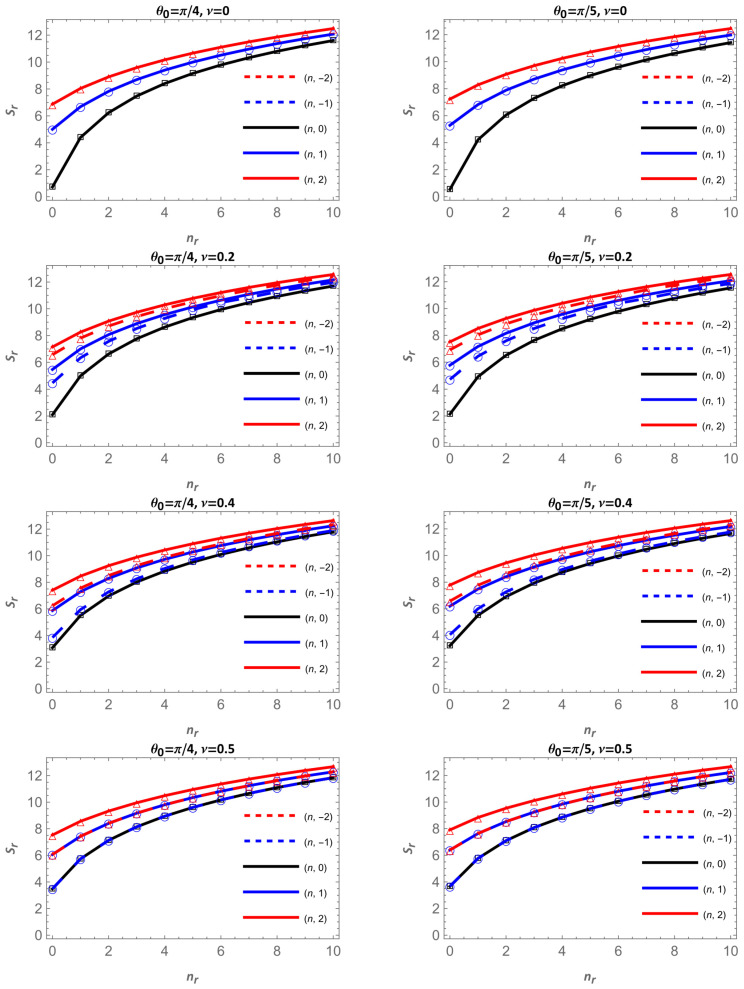
Shannon entropy in configuration space as a function of $n_{r}$ for $\theta_{0}=\pi /4\;and\;\pi /5$. Continuous lines correspond to m=0 in black, m=1 in blue and m=2 in red; dashed lines are for m=−2, in red, and for m=−1 in blue.

Figure 9 presents the Shannon entropy in configuration space, $S_{r}$, as a function of the radial quantum number $n_{r}$ for the apex angles $\theta_{0}=\pi /2$ (left column) and $\theta_{0}=\pi /3$ (right column). In each column, the magnetic flux increases downward through the values ν=0, 0.2, 0.4 and 0.5. The solid curves correspond to m≥0, black for m=0, blue for m=1, and red for m=2, while dashed curves represent their negative counterparts. Figure 10 shows the same quantity for the other two apex angles, $\theta_{0}=\pi /4$ and π/5 ; following the same organization, each column corresponds to one fixed angle, and the magnetic flux increases from top to bottom.

For all cases, $S_{r}$ increases with the radial quantum number $n_{r}$, confirming that higher excited states are more spatially extended, as anticipated from the broadening of the radial probability densities. However, the dependence on the cone angle $\theta_{0}$ and on the magnetic flux ν reveals a more intricate behavior that depends on the azimuthal quantum number m; a similar behavior was observed for an electron on the surface of a cone in the presence of the Aharonov–Bohm field.

For the states $\left(n_{r},m\right)$ = $\left(n_{r},0\right)$ and ν=0, the entropy $S_{r}$ decreases as the cone becomes narrower, i.e., as $\theta_{0}$ is reduced from $\theta_{0}=\pi /2\;to\;\pi /5$, as can be seen in Table 1. This is a purely geometric (spatial confinement) effect: for $\left(n_{r},m\right)$ = $\left(0,0\right)$ and ν=0 there is no centrifugal barrier, and the only source of confinement is the conical geometry itself. As the apex angle decreases, the available surface area is reduced, and the probability density becomes more concentrated near the apex, leading to a lower spatial uncertainty and consequently smaller $S_{r}$. This geometric localization explains why, in the black curves of all columns, the entropy systematically decreases with decreasing $\theta_{0}$ in the absence of magnetic flux.

In contrast, when m≠0 or a finite magnetic flux is present (Table 2 and Table 3), the overall trend of $S_{r}$ with respect to the angle $\theta_{0}$ becomes more intricate. For states with low radial quantum numbers, the centrifugal term associated with the angular momentum, through the effective parameter $\mu =\left|m+\nu \right|/sin\;\theta_{0}$, dominates over the geometric confinement. As $sin\;\theta_{0}$ decreases, μ increases, pushing the probability density toward larger radii and producing a more extended distribution, which results in higher values of $S_{r}$. However, as $n_{r}$ increases, the influence of μ weakens compared with the purely geometric effect, and $S_{r}$ decreases again as the cone narrows, as can be seen in Table 1, Table 2 and Table 3. Altogether, these results show that the competition between geometric confinement and the centrifugal effect, enhanced by the Aharonov–Bohm flux, governs the non-monotonic dependence of $S_{r}$ on the apex angle, leading to either localization or delocalization depending on the state.

Along each column in Figure 9 and Figure 10, as the magnetic flux ν increases downward, distinct trends appear, depending again on the sign of m. For m≥0 (solid curves), $S_{r}$ grows gradually with ν. Increasing ν enhances $\left|m+\nu \right|$, thus increasing μ and expanding the radial probability density outward, leading to a broader, less localized distribution, consistent with higher entropy values. On the other hand, for negative m (dashed curves), the entropy decreases with increasing ν. In this case, raising the flux reduces $\left|m+\nu \right|$, decreasing the effective angular-momentum barrier, which compresses the density closer to the apex and lowers. These opposite behaviors, an upward trend for m≥0 and a downward one for m<0, are clearly visible across all four angles.

Altogether, Figure 9 and Figure 10 show that the Shannon entropy in configuration space is governed by the interplay between geometry, magnetic flux, and angular momentum. The purely geometric behavior occurs only for the states $\left(n_{r},m=0\right)$ with ν=0, where the confinement imposed by the conical surface dominates and the entropy decreases as the cone becomes narrower. In all other cases, when the system carries angular momentum or a finite magnetic flux, the effective parameter $\mu =|m+\nu |⁄(sin\;\theta_{0})\;$ introduces a centrifugal-like barrier that counteracts the geometric confinement. Under these conditions, the combined centrifugal–magnetic effect can increase $S_{r}$ as the apex angle decreases, particularly for low-lying states where the influence of μ is stronger. However, for higher radial excitations, the geometric effect regains dominance and $S_{r}$ tends to decrease again as the cone narrows. These trends reveal a dynamic competition between confinement and delocalization that depends on both the angle and the quantum state, and are consistent with the results obtained for the energies and radial densities.

#### 4.3.2. Shannon Entropy in Momentum Space

**Figure 11:**
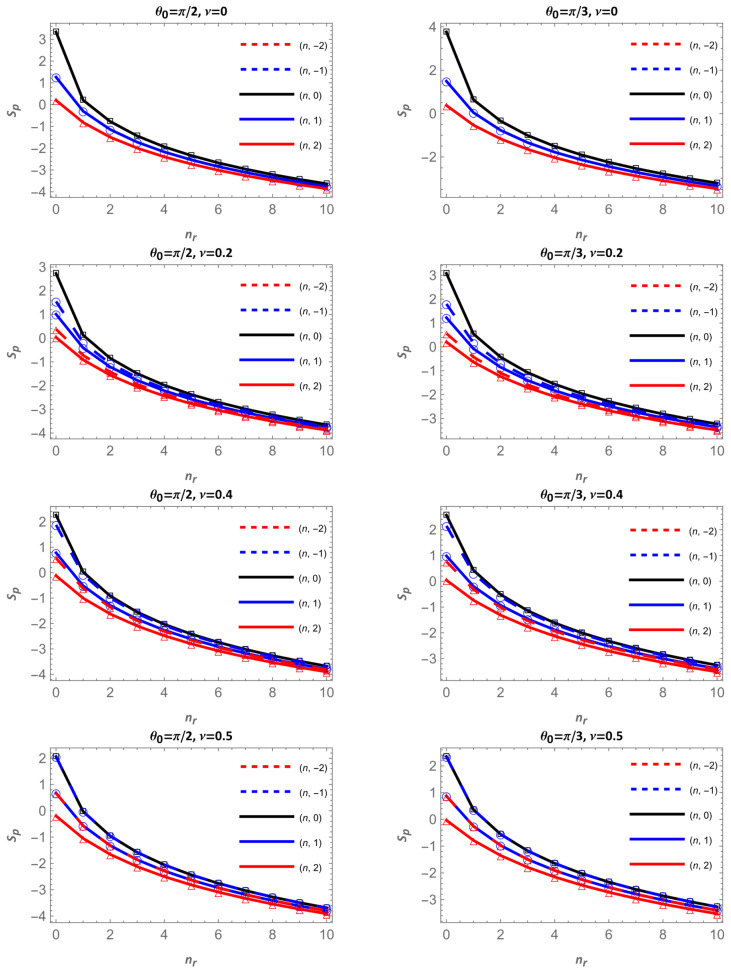
Shannon entropy in momentum space as a function of $n_{r}$ for $\theta_{0}=\pi /2\;and\;\pi /3$. Continuous lines correspond to m=0 in black, m=1 in blue and m=2 in red; dashed lines are for m=−2, in red, and for m=−1 in blue.

**Figure 12:**
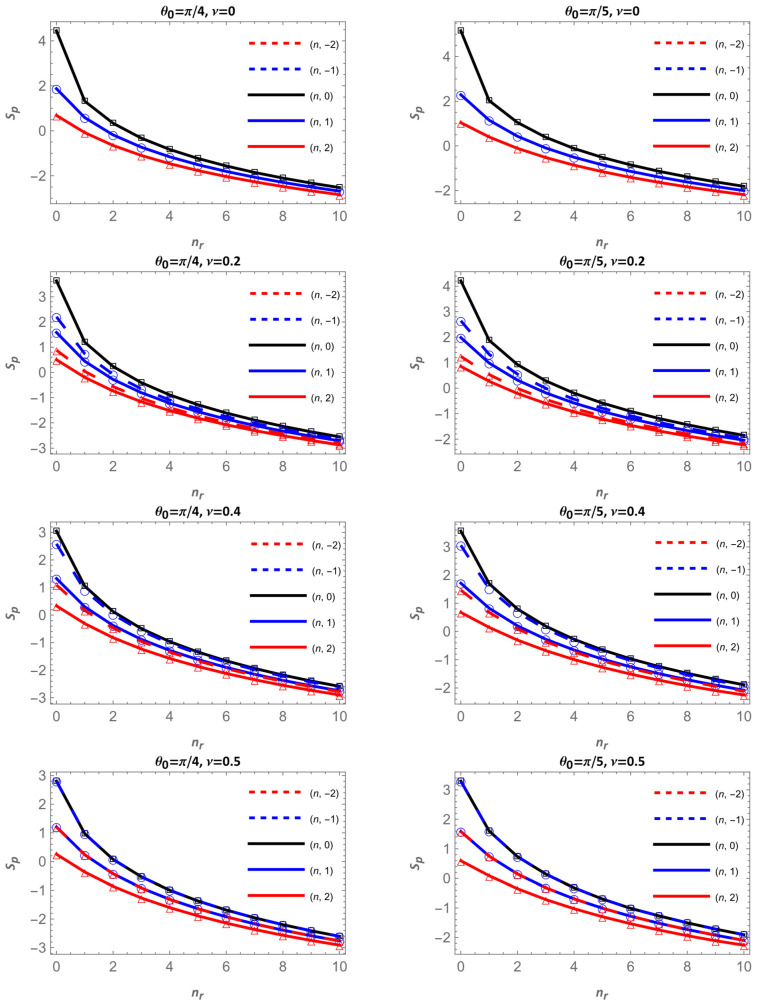
Shannon entropy in momentum space as a function of $n_{r}$, for $\theta_{0}=\pi /4\;and\;\pi /5$. Continuous lines correspond to m=0 in black, m=1 in blue and m=2 in red; dashed lines are for m=−2, in red, and for m=−1 in blue.

Figure 11 and Figure 12 display the Shannon entropy in momentum space $S_{p}$ for different apex angles $\theta_{0}$ and magnetic flux values ν. Each figure contains two columns: $\theta_{0}=\pi /2\;and\;\pi /3$ in Figure 11 and $\theta_{0}=\pi /4\;and\;\pi /5$ in Figure 12. The magnetic flux increases downward ν=0, 0.2, 0.4 and 0.5.

Overall, $S_{p}$ increases as the cone becomes narrower, that is, when $\theta_{0}$ decreases. For a given flux and angular momentum, the curves corresponding to smaller apex angles lie above those of larger angles, indicating that narrower cones generate a broader momentum-space distribution and hence a higher entropy. This effect is more pronounced for the lowest radial states, where the geometry strongly shapes the wavefunction profile.

Regarding the magnetic flux, the behavior is dual and depends on the sign of m. For positive m values (solid lines), $S_{p}$ decreases as the ν increases. In this case, increasing ν enhances $\left|m+\nu \right|$, strengthening the angular confinement and reducing the spread of the wavefunction in momentum space. Conversely, for negative m values (dashed lines), $S_{p}$ increases with ν, because $\left|m+\nu \right|$ becomes smaller, the confinement weakens, and the momentum distribution broadens, leading to larger entropy values. This opposite trend between solid and dashed curves is observed across all apex angles.

Finally, unlike in configuration space, $S_{p}$ decreases with the radial quantum number $n_{r}$ since in this case the wavefunction becomes more extended in real space, and its momentum-space representation becomes more concentrated, resulting in lower uncertainty in momentum. Taken together, Figure 11 and Figure 12 confirm the complementary behavior between $S_{r}$ and $S_{p}$: under the same conditions, stronger geometric or magnetic confinement reduces entropy in configuration space, leading to an opposite variation in momentum space and preserving the entropic balance of the system.

#### 4.3.3. Shannon Entropy Sum

As shown in the Shannon entropy figures in both configuration and momentum space, when one increases, the other decreases. The BBM uncertainty principle for the present two-dimensional (*D* = 2) problem is reduced to

$$S_{t}=S_{r}+S_{p}\geq 2\left(1+\ln\pi \right)=4.2896.$$

**Figure 13:**
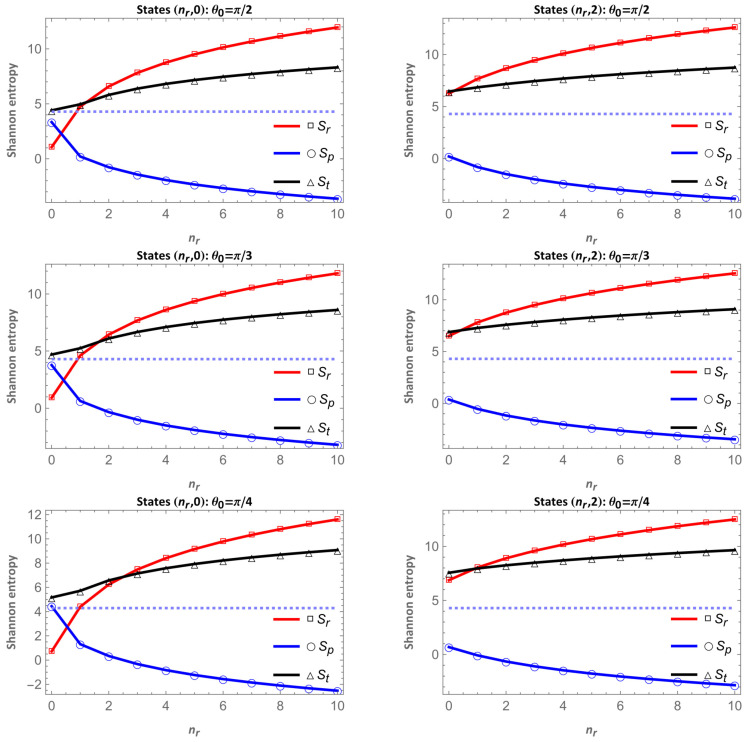
Entropic sum as a function of $n_{r}$ for the states ($n_{r}$,0) and ($n_{r}$,2), for different values of the apex angle $\theta_{0}$ and ν=0. The dotted line corresponds to the value of the bound of the BBM uncertainty principle; $S_{t}=S_{r}+S_{p}\geq 2\left(1+\ln\pi \right)=4.2896$, as expected.

We calculated this entropic sum for each state, angle and magnetic flux presented in this study, and we found that the BBM uncertainty principle is satisfied in all cases, as can be seen in Figure 13.

The plots of the total Shannon entropy $S_{t}=S_{r}+S_{p}$ show that, as the radial quantum number $n_{r}$ increases, the entropy in configuration space $S_{r}$ rises while the corresponding momentum-space entropy $S_{p}$ decreases. This opposite behavior reflects the complementarity between both representations and explains why the sum $S_{t}$ is not constant but exhibits a moderate increase with $n_{r}$. The lack of perfect compensation between $S_{r}$ and $S_{p}$ arises because the redistribution of probability between configuration and momentum spaces is not completely symmetric; the broadening of the spatial density does not generate an equivalent contraction in momentum space.

In all analyzed cases, the black curve representing $S_{t}$ remains clearly above the theoretical lower bound $\;S_{t}\geq 2\left(1+\ln\pi \right)$, confirming the fulfillment of the Bialynicki-Birula–Mycielski inequality and the internal consistency of the numerical results. The fact that $S_{t}$ is not a constant but slightly increases with the radial excitation or with the combined influence of geometry and angular momentum indicates that the overall complexity of the system grows as the electronic distribution becomes more extended and structurally richer.

## 5. Conclusions

In this work, we have investigated the behavior of a hydrogenic atom confined to the surface of a right circular cone under the influence of an Aharonov–Bohm magnetic flux. The combined analysis of energy spectra, probability densities, and Shannon entropies in both configuration and momentum spaces enabled us to precisely determine how geometry and magnetic flux modify the system’s quantum structure and information distribution.

The energy spectra exhibit a clear dependence on both the apex angle and the magnetic flux. As the cone becomes narrower or the flux increases, the energy levels shift and their spacing decreases, reflecting the strengthening of the effective confinement. In particular, the Aharonov–Bohm flux modifies the system’s boundary conditions and breaks the symmetry between states with m and −m, shifting their energies in opposite directions. This effect also leads to periodic degeneracies at fractional flux values, such as ν=±0.5, in full agreement with the theory of cylindrically symmetric systems under a confined magnetic flux. This same effect is observed in the system of an electron, in the absence of a positively charged nucleus, moving on the surface of a cone in the presence of an Aharonov–Bohm magnetic flux [50].

The radial probability densities confirm the joint influence of geometry and flux. Narrower cones and finite magnetic flux redistribute the electron probability toward larger radii, thereby reducing the likelihood of finding the particle near the apex. The Aharonov–Bohm flux acts as a fine-tuning parameter for radial confinement: depending on the sign of m, it can either increase or decrease the effective barrier $\mu =\left|m+\nu \right|/sin\;\theta_{0}$, modulating both localization and delocalization in a controllable way.

The entropic analysis complements and reinforces these findings. The Shannon entropies $S_{r}$ and $S_{p}$ exhibit reciprocal behaviors that reflect the redistribution of probability between both spaces, while the total entropy $S_{t}$ always remains above the theoretical lower bound $S_{t}\geq 2\left(1+\ln\pi \right)$, confirming the validity of the entropic uncertainty principle. The fact that it is not constant, slightly increasing with radial excitation, magnetic flux, or angular momentum, indicates that the overall complexity of the system increases as the electronic distribution becomes more extended and structurally richer.

Overall, the results reveal apparent coherence among the system’s energetic, spatial, and entropic behaviors. The interplay between the conical geometry and the Aharonov–Bohm flux precisely determines the degree of confinement and the electronic probability distribution, highlighting the close connection between the spectral structure and the information-theoretic properties of bound states on conical surfaces.

## Figures and Tables

**Table 1:** Values of the Shannon entropy $S_{r}$ for m=0, ν=0, and different apex angles $\theta_{0}$.

	$S_{r}:m=0,\nu =0$			
$n_{r}$	θ=π/2	θ=π/3	θ=π/4	θ=π/5
0	1.0653	0.92145	0.7187	0.5339
1	4.7461	4.6022	4.3995	4.2147
2	6.5843	6.4404	6.2377	6.0529
3	7.8203	7.6764	7.4737	7.2889
4	8.7535	8.6097	8.4069	8.2221
5	9.5039	9.3601	9.1573	8.9725
6	10.1317	9.9879	9.7852	9.6003
7	10.6716	10.5278	10.3250	10.1402
8	11.1452	11.0014	10.7987	10.6139
9	11.5672	11.4234	11.2207	11.0358
10	11.9477	11.8039	11.6012	11.4163

**Table 2:** Values of the Shannon entropy $S_{r}$ for m=1, ν=0, and different apex angles $\theta_{0}$.

	$S_{r}:m=1,\nu =0$			
$n_{r}$	θ=π/2	θ=π/3	θ=π/4	θ=π/5
0	4.5420	4.7227	5.0045	5.2875
1	6.5097	6.5711	6.6873	6.8238
2	7.7817	7.7893	7.8262	7.8881
3	8.7299	8.7064	8.6962	8.7122
4	9.4880	9.4441	9.4025	9.3872
5	10.1203	10.0620	9.9979	9.9599
6	10.663	10.5939	10.5129	10.4576
7	11.1385	11.0611	10.9669	10.898
8	11.5618	11.4778	11.373	11.2931
9	11.9433	11.8538	11.7403	11.6514
10	12.2905	12.1965	12.0757	11.9791

**Table 3:** Values of the Shannon entropy $S_{r}$ for m=−1, ν=0.2, and different apex angles $\theta_{0}$.

	$S_{r}:m=-1,\nu =0.2$			
$n_{r}$	θ=π/2	θ=π/3	θ=π/4	θ=π/5
0	4.0722	4.2265	4.4729	4.7258
1	6.2243	6.2599	6.3382	6.4395
2	7.5750	7.5606	7.5642	7.5937
3	8.5675	8.5251	8.4858	8.4729
4	9.3540	9.2937	9.2265	9.1853
5	10.0062	9.9334	9.8465	9.7851
6	10.5636	10.4816	10.3800	10.3035
7	11.0504	10.9614	10.8485	10.7601
8	11.4828	11.3881	11.2661	11.1683
9	11.8716	11.7723	11.643	11.5374
10	12.2249	12.1218	11.9863	11.8743

## Data Availability

This manuscript has no associated data, or the data will not be deposited. The data that support the findings of this study are available from the corresponding author upon reasonable request.

## References

[1] Frieden B.R. (1998). Physics from Fisher Information: A Unification.

[2] Shannon C. (1948). A mathematical theory of communication. Bell Syst. Tech. J..

[3] Cover T.M., Thomas J.A. (2006). Elements of Information Theory.

[4] Huang J., Supaongprapa T., Terakura I., Wang F., Ohnishi N., Sugie N. (1999). A model-based sound localization system and its application to robot navigation. Robot. Auton. Syst..

[5] Philippatos G., Wilson C. (1972). Entropy, market risk, and the selection of efficient portfolios. Appl. Econ..

[6] Huang X. (2008). Mean-entropy models for fuzzy portfolio selection. IEEE Trans. Fuzzy Syst..

[7] Yang L. (2012). Study on cumulative residual entropy and variances as risk measure. 2012 Fifth International Conference on Business Intelligence and Financial Engineering.

[8] Wu S., Wang S. (2011). Information-theoretic outlier detection for large-scale categorical data. IEEE Trans. Knowl. Data Eng..

[9] Das D., Zhou S. (2017). Detecting entropy increase in categorical data using maximum entropy distribution approximations. IISE Trans..

[10] Prato M., Zanni N. (2008). Inverse problem in machine learning: An application to brain activity interpretation. J. Phys. Conf. Ser..

[11] Watanabe S. (1981). Pattern recognition as a quest for minimum entropy. Pattern Recognit..

[12] Llenas A., Riihijarvi J., Petrova M. (2017). Performance evaluation of machine learning based signal classification using statistcal and multiscale entropy features. 2017 IEEE Wireless Comunication and Networking Conference (WCNC).

[13] Shwartz R., LeCun Y. (2024). To compress or Not to compress-Self-supervised Learning and information theory: A Review. Entropy.

[14] Wang Y. (2016). Seismic Inversion: Theory and Applications.

[15] Gull S., Skilling J. (1984). Maximum entropy method in image processing. IEE Proceedings F (Communications, Radar and Signal Processing).

[16] Wu Y., Zhou Y., Saveriades G., Agaian S., Noonan J.P., Natarajan P. (2013). Local Shannon entropy measure with statistical test for image randomnes. Inf. Sci..

[17] Sparavigna A. (2015). On the role of Tsallis entropy in image processing. Int. Sci. Res. J..

[18] Bertero M., Plana M. (2006). Inverse Problems in Biomedical Imaging: Modeing and Methods of Solution.

[19] Gao J., Hu J., Tung W. (2012). Entropy measures for biological signal analyses. Nonlinear Dyn..

[20] Grasshans F., Cerf N.J. (2004). Continuous-Variable Quantum Cryptography is Secure against Non-Gaussian Attacks. Phys. Rev. Lett..

[21] del Río F., López-Hernández R., Chaparro C. (2024). On information, entropy and early stone tools. Mol. Phys..

[22] Casadio R., da Rocha R., Meert P., Tabarroni L., Barreto W. (2023). Configurational entropy of black hole quantum cores. Class. Quantum Grav..

[23] Dehesa J.S., Belega E.D., Toranzo I.V., Apterakev A.I. (2019). The Shannon entropy of high-dimensional hydrogenic and harmonic systems. Int. J. Quantum Chem..

[24] Bouvrie A., Angulo J.C., Dehesa J.S. (2011). Entropy and complexity analysis of Dirac-delta-like quantum potentials. Phys. A.

[25] Sun G.H., Aoki M.A., Dong S.H. (2013). Quantum information entropies of the eigenstates for the Pöschl-Teller-like potential. Chin. Phys. B.

[26] Hô M., Weaver D.F., Smith V.H., Sagar R.P., Esquivel R.O. (1998). Calculating the logarithmic mean excitation energy from the Shannon information entropy of the electronic charge density. Phys. Rev. A.

[27] Karafiloglou P., Panos C.P. (2004). Order of Coulomb and Fermi pairs: Application in a π-system. Chem. Phys. Lett..

[28] Maroulis G., Sana M., Leroy G. (1981). Molecular properties and basis set quality: An approach based on information theory. Int. J. Quantum Chem..

[29] Simas A.M., Thakkar A.J., Smith V.H. (1983). Basis set quality. II. Information theoretic appraisal of various s-orbitals. Int. J. Quantum Chem..

[30] Grade S.R., Sears S.B., Chakravorty S.J., Bendale R.D. (1985). Some novel characteristics of atomic information entropies. Phys. Rev. A.

[31] Chen Z., Wannere C.S., Corminboeuf C., Putcha R., Schleyer P.V.R. (2005). Nucleus-independent chemical shifts (NICS) as an aromaticity criterion. Chem. Rev..

[32] Hô M., Sagar R.P., Pérez J.M., Smith V.H., Esquivel R.O. (1994). A numerical study of molecular information entropies. Chem. Phys. Lett..

[33] Nagy A., Parr R.G. (1996). Information entropy as a measure of the quality of an approximate electronic wave function. Int. J. Quantum Chem..

[34] Prasad V., Yadav C., Vidhani B., Arora M., Tyagi A., Dahiya B. (2024). Quantum entropic exchangeat avoided crossing due to laser-atom interaction. Physica A.

[35] Kumar K., Prasad V. (2025). Analysis of Shannon entropy and Quantum states of a confined hydrogen atom screened by the Hellmann potential. Adv. Theory Simul..

[36] Joshi R., Verma N., Mohan M. (2023). Shannon entropy along hydrogen isoelectric sequence using numerov method. Rev. Méx. Fís..

[37] Savirov D., Koledina K. (2020). Classification of isentropic molecules in terms of Shannon entropy. EPJ Web Conf..

[38] Sen K.D. (2005). Characteristic features of Shannon information entropy of confined atoms. J. Chem. Phys..

[39] Martínez-Sánchez M.A., Vargas R., Garza J. (2019). Shannon entropy for the hydrogen atom confined by four different potentials. Quantum Rep..

[40] Rodríguez-Bautista M., Vargas R., Aquino N., Garza J. (2017). Electron-density delocalization in many-electron atoms confined by penetrable walls: A Hartree–Fock study. Int. J. Quantum Chem..

[41] Martínez-Flores C. (2021). Shannon entropy and Fisher information for endohedral confined one and two-electron atoms. Phys. Lett. A.

[42] Nascimento W.S., Prudente F.V. (2016). Sobre um estudo da entropia de Shannon no contexto da mecânica quântica: Uma aplicação ao oscilador harmônico livre e confinado. Quim. Nova.

[43] Corzo H.H., Castaño E., Laguna H.G., Sagar R.P. (2013). Measuring localization-delocalization phenomena in a quantum corral. J. Math. Chem..

[44] Song X.D., Sun G.H., Dong S.H. (2015). Shannon information entropy for an infinite circular well. Phys. Lett. A.

[45] Shafeekali H., Olendski O. (2023). Quantum-information theory of magnetic field influence on circular dots with different boundary conditions. Phys. Scr..

[46] Estañón C.R., Montgomery H.E., Angulo J.C., Aquino N. (2024). The confined helium atom: An informational-theoretic approach. Int. J. Quantum Chem..

[47] Majumdar S., Roy A.K. (2020). Shannon entropy in confined helium-like ions within a Density Functional formalism. Quantum Rep..

[48] Nasser I., Zeama M., Abdel-Hady A. (2020). Rényi, Fisher, Shannon and their electron correlation tools for two-electron series. Phys. Scr..

[49] Nasser I., Abdel-Hady A. (2020). Fisher information and Shannon entropy calculations for two-electron systems. Can. J. Phys..

[50] Arvizu L.M., Castaño E., Aquino N. (2025). Shannon entropy of an electron on a conical surface: Aharonov-Bohm effects. J. Phys. A Math. Theor..

[51] Arvizu L.M., Castaño E., Aquino N. (2024). Shannon entropy of a particle on a conical surface. Phys. Scr..

[52] Olendski O. (2019). Quantum information measures of the Aharonov-Bohm ring in uniform magnetic fields. Phys. Lett. A.

[53] Olendski O. (2022). Quantum-information theory of a Dirichlet ring with Aharonov-Bohm field. Eur. Phys. J. Plus.

[54] Omugbe E., Osafile O.E., Njoku I.J., Jahanshir A., Edet C.O., Okon I.B., Eyube E.S., Onate C.A., Horchani R., William E.S. (2023). Information-theoretic measures and thermodynamic properties under magnetic and Aharonov-Bohm flux fields. Eur. Phys. J. D.

[55] Lima F.C.E., Moreira A.R.P., Almeida C.A.S., Edet C.O., Ali N. (2023). Quantum information entropy of a particle trapped by the Aharonov-Bohm-type effect. Phys. Scr..

[56] Sargsian T.A., Mkrtchyan M.A., Sarkisyan H.A., Hayrapetyan D.B. (2021). Effects of the external electric and magnetic fields of the linear and nonlinear optical properties of InAs cylindrical quantum dot with modified Poschl-Teller and Morse confinement potentials. Phys. E Low-Dims. Syst. Nanostruct..

[57] Kirak M., Yilmaz S., Sahin M., Gencaslan M. (2011). The electric field effects on binding energies and nonlinear optical properties of donor impurity in spherical quantum dot. J. Appl. Phys..

[58] Xie W. (2009). Absorption spectra of a donor impurity in quantum ring. Phys. Status Solidi.

[59] Xie W. (2012). Optical anisotropy of donor in ellipsoidal dots. Phys. B Condens. Matter.

[60] Hayrapetyan D.B., Kazaryan E.M., Sarkisyan H.A. (2016). Magneto-absorption in conical quantum dot ensemble: Possible applications for QD LED. Opt. Commun..

[61] Lozovski V., Piatnytsia V. (2011). The analytical study of electronic and optical properties of pyramid-like and cone-like quantum dots. J. Comput. Theor. Nanosci..

[62] Aquino N., Castaño E., Arvizu L.M., Flores-Riveros A. (2026). Hydrogenic atom on the surface of a cone. Phys. Scr..

[63] Yang X.L., Guo S.H., Chan F.T., Wong K.W., Ching W.Y. (1991). Analytical solution of a two-dimensional hydrogen atom. I Nonrelativistic theory. Phys. Rev. A.

[64] Aquino N., Castaño E. (1998). Confinement effects in hydrogen-like two-dimensional atoms. Rev. Mex. Fís..

[65] Bialynicki-Birula I., Mycielski J. (1975). Uncertainty relations for information entropy in wave mechanics. Commun. Math. Phys..

